# Fat feeding potentiates the diabetogenic effect of dexamethasone in Wistar rats

**DOI:** 10.1186/1755-7682-1-7

**Published:** 2008-05-23

**Authors:** Shanmugam Sivabalan, Shanmugam Renuka, Venugopal P Menon

**Affiliations:** 1 Department of Biochemistry & Biotechnology, Faculty of Science, Annamalai University, Annamalainagar – 608002, Tamilnadu, India

## Abstract

**Background:**

The role of cortisol and its increased action/availability is implicated in the pathogenesis of insulin resistance associated with obesity and metabolic syndrome but the mechanism of increased action/availability is not known. Availability of several other lipophilic hormones, drugs and pollutants are also reported to be increased in obesity. Increased lipids in the circulation are reported to alter the fluidity and permeability of membranes. Hyperlipidemia is also reported to alter the pharmacokinetics and pharmacodynamics of lipophilic molecules and also membrane fluidity and permeability. In this context we assumed that the hyperlipidemia associated with human obesity might play a role in the altered action/availability of cortisol and this in turn might have initiated the metabolic complications. To evaluate our assumption we have administered dexamethasone [low [50 μg/kg/day] or high [250 μg/kg/day] dose] to high-fat [coconut oil & vanaspati] fed rats and the results were compared with rats administered with either dexamethasone or high-fat.

**Results and Discussion:**

Within two weeks, the rats co-administered with high-fat and dexamethasone developed severe hyperglycemia, hyperlipidemia and insulin resistance compared to rats treated either of them alone. High-fat fed rats treated with higher dose of dexamethasone were presented with severe hyperglycemia, insulin resistance and also severe glycosuria. The hyperlipidemia caused by high-fat feeding might have altered the transport and distribution of dexamethasone, probably by altering the physical state of membranes and transport proteins.

**Conclusion:**

From the results obtained, it can be speculated that the altered lipid and cortisol metabolism could affect one another, forming a vicious cycle.

## Background

Type 2 diabetes, obesity and metabolic syndrome are emerging at an alarming rate worldwide [1-3]. Developing societies worldwide shifting away from an agrarian existence to the current environment of high energy consumption, minimal physical activity and a lifestyle that include stress and anxiety are some of the several factors implicated in the development of these disorders [4-8]. The fundamental aspect in the etiology of these disorders is insulin resistance and it is linked to a wide array of other complications including gestational diabetes and polycystic ovarian syndrome [3,9,10]. Insulin resistance is a disorder in which target cells fail to respond to ordinary levels of circulating insulin, hence higher than normal concentrations of insulin are needed in order to maintain normoglycemia. In response, more and more insulin is produced by the pancreas, leading to hyperinsulinemia. The main characteristics of insulin resistance are uncontrolled lipolysis in adipose tissue, impaired uptake of glucose by muscle and uncontrolled gluconeogenesis in the liver. Over time, β cell compensation for the insulin resistance fails, resulting in a progressive decline of β cell function which leads to type 2 diabetes [3,11]. In the past decade, a large number of endocrine, inflammatory, neural, cell-intrinsic pathways and fatty acid composition/metabolism have been shown to be dysregulated in obesity causing insulin resistance [6,12,13]. Although it is possible that one of these factors plays a dominant role, many of these factors are interdependent, and it is likely that their dynamic interplay underlies the pathogenesis of insulin resistance. Understanding the biology of these systems will inform the search for interventions that specifically prevent or treat insulin resistance and its associated pathologies [12].

Increased free fatty acid [FFA] concentration, whether due to increased endogenous mobilization or post absorptive lipolysis on a fat-rich diet, is associated with insulin resistance and β cell dysfunction, making them a likely culprit [13,14]. There is now growing evidence that the original theory [Randle or glucose-fatty acid cycle] supposing that the decreased glucose uptake in muscle is a result of increased fatty acid oxidation and suppressed glucose oxidation cannot completely explain all experimental data [15,16]. The metabolic defect reported involves early insulin signaling pathways and is independent of FFA oxidation [17]. Fatty acid composition in the diet is another mechanism implicated in the development of insulin resistance [6]. Replacing saturated fatty acids in the diet with either monounsaturated fatty acids or polyunsaturated fatty acids resulted in changes in serum fatty acid profile and improved insulin sensitivity [18,19]. This improvement in insulin sensitivity was found particularly only in subjects with a relatively low fat intake [below median 37% energy] [18,19]. Recent reports suggest that the role of increased lipids in the development to type 2 diabetes is a secondary phenomenon, pathogenesis of diabetes primarily established by involving genetic and environmental factors [20].

Several environmental factors, including high-fat diet, are reported to activate the functioning of the hypothalamus-pituitary-adrenal axis [HPA]. Frequently evoked HPA-axis secretes excessive amount of cortisol [8,21] and elevated cortisol level is implicated in the development of entire spectrum of the metabolic syndrome, including insulin resistance, visceral obesity and dyslipidemia as well as the kinds of cardiovascular co morbidities that result [8,21,22].

Half a century ago, Jean Vague have suggested that the role of over activity of pituitary-adrenal axis, greater abundance/stronger action of cortical steroids and its anti-insulin effect, in the development of android [visceral] obesity and its complication while gynoid obesity is free of these effects and the circulation of lipids is also effected more slowly [23]. Now there are several studies suggest that frequent stress or perturbed secretion of cortisol in the development of visceral obesity, insulin resistance and its pathologies [8,21]. Glucocorticoids bring about their multiple effects by activating the intracellular glucocorticoids receptor that binds to specific glucocorticoids-responsive elements in the vicinity of regulated genes and subsequently affect their expression. It is estimated that glucocorticoid receptors can interact as transcription factors as many as 30% of genes, so it is not surprising that glucocorticoids induce a wide range of responses [24]. Glucorticoids oppose the insulin-mediated inhibition of hepatic glucose release [i.e. stimulate gluconeogenesis] and decrease glucose use in muscle [4].

Although there are several reports suggest the role of glucocorticoids in the development of insulin resistance and its complications, its path physiological contribution to idiopathic human obesity and its associated metabolic complications has been the subject of long debate, dominated by discussion of the inconsistent changes that occur in plasma cortisol levels [25]. This controversy is explained by the increased clearance and increased intracellular action/availability of cortisol independent of its plasma levels [26-28]. The altered level or action of cortisol in obesity have been explained by the altered expression of the enzyme 11 β-hydroxysteroid dehydrogenase type 1 [11 β-HSD1] in adipose tissue which generate cortisol from inactive corticosterone [28], but the levels of expression and activity of 11 β-HSD1 are debated in the literature [28,29]. And this enzyme is exquisitely regulated including by feeding, and it might be a key component in the homeostatic adaptation to variations in macronutrient intake [24,30]. Although the role of cortisol and its increased clearance, action/availability are generally accepted in the pathogenesis insulin resistance [8,26-28,30], the mechanism involved in the altered action/availability is not known.

Apart from cortisol, several other sex steroids are also reported to be decreased in obesity or its clearance is reported to be increased [31-33]. It is well known that lipophilic molecules tend to accumulate in fat organs, a phenomenon called "bioaccumulation" [5]. This have been demonstrated using the unmetabolizable lipophilic compound [2,4,5,2',4' 5'-Hexachlorobiphenyl [6-CB] – a polychlorinated biphenyl] in rats [34]. Once administered, this compound disappears with a half-life of half a life span of the rats by fecal excretion and its accumulation is proportional to the adipose tissue mass. Although unmetabolizable lipophilic molecules tend to accumulate, the metabolizable lipophilic-drugs are prevented from accumulation by intracellular drug metabolizing enzymes [34]. It is not known whether this theory is applied to exogenously administered or endogenously produced lipophilic steroid hormones.

Studies have shown that changes in lipid profile [i.e. caused by hormonal variations, weight gain, diet, medication, illness & including normal biological fluctuations] alter the phamacodynamic and pharmacokinetic of lipophilic drugs [35-37]. A recent study has shown that the pharamacological activity of the lipophilic drug [Clozapine] is potentiated by hyperlipidemia, by facilitating its blood brain barrier permeability [35].

Several reports have also shown that altered cortisol metabolism affect lipid metabolism and altered lipid metabolism affect cortisol metabolism [4,30], vice versa. But it is not known whether altered cortisol metabolism precede the obese state related complications or excess body fat precede the altered cortisol metabolism [22]. Most of the available works deals these two states such as altered cortisol metabolism [8,21] and altered lipid metabolism [13] as two different etiological factors in the development of obesity, insulin resistance and its related disorders. But these two factors are associated in several pathological conditions such as obesity, metabolic syndrome, gestational diabetes, polycystic ovarian syndrome and type 2 diabetes [9,10,26,38].

Though glucocorticoids are helpful in adapting to short-term stress, excessive glucocorticoid action becomes maladaptive, particularly if this excessive action occurs in the absence of starvation [24]. This have been demonstrated in rats administered parenteral nutrition, that the insulin resistance caused by Dex is reduced significantly when the fat/calorie content of parenteral nutrition is reduced to 50% of the adequate level [39]. Modern lifestyle increases the likelihood of individuals eating more than they need and the affluent sedentary lifestyle leads to sustained positive caloric balance [3]. In many of the developed societies, there is also a high level of perceived stress [8]. The simultaneous presence of the sustained increase of lipids in the circulation and chronic/repeated stress induced secretion of cortisol is very much possible in the modern world [8]. Cortisol is a lipophilic hormone [40] and obesity is mostly associated with dysregulation of lipid metabolism [3]. From these observations we have assumed that the hyperlipidemia associated with positive caloric balance or obesity might have facilitated the intracellular action/availability of cortisol, which in turn might have involved in the pathogenesis of insulin resistance and its related disorders.

So we planned to study the combined effect of altered lipid and cortisol metabolism, to know if there is any synergistic or interaction between the two, and their effect in the development of insulin resistance. To know the combined effect of altered lipid and cortisol metabolism, we have administered the cortisol analog [Dexamethasone] Dex to high-fat [coconut oil & vanaspati] fed Wistar rats. Dex has been administered at varying dose and duration by several studies to induce insulin resistance [41,42]. We have selected two doses of Dex [low dose: 50 μg/kg/day [41] and a high dose: 250 μg/kg/day] in our study. We have used coconut oil [43] and Indian vanaspati [44] to induce hyperlipidemia, as they are good source of saturated and trans fatty acids. To control the daily intake of fat, we have administered the high-fat via gavage [45]. We planned to continue the treatment for 12 weeks, but surprisingly, all the high-fat fed rats treated with higher dose of Dex [250 μg/kg/day] developed severe glycosuria and polyuria on fourteenth day of treatment. During the experimental days we have analyzed the body weight, food and water intake, and upon seeing the glycosuria on 14 day, we have analyzed the lipid profile, insulin resistance and oral glucose tolerance test. The combined effects of Dex and high-fat in the development of insulin resistance and hyperglycemia were correlated with its individual effects and the results were related with other similar pathological and experimental situations.

## Methods

### Animals

Male Albino rats, Wistar strain of body weight ranging 250–300 g bred in Central Animal House, Rajah Muthiah Medical College, Tamilnadu, India, fed on laboratory diet [Agro Corporation Private Limited, Bangalore, India] were used for the study. Water and food was given ad libitum. The standard pellet diet comprised 21% protein, 5% lipids, 4% crude fiber, 8% ash, 1% calcium. 0.6% phosphorus, 3.4% glucose, 2% vitamin and 55% nitrogen free extract [carbohydrates]. The fatty acid composition of vanaspati is follows: 14:0-0.9, 16: 0-38.5, 18: 0-5.2, 18:1 trans n-9-16.2, 18:1 cis 34.0 and 18:2 n-6-5.0 and the fatty acid composition of coconut oil is as follows: 14:0-48.9, 16:0-21.96, 18:0-6.0, 18:1-18.2, 18:2 [ω-6]-5.33 [43,44].

The animals were housed in plastic cages under controlled conditions of 12 h light/12 h dark cycle and at 24 ± 2 °C. The animals used in the present study were maintained in accordance with the guidelines of the National Institute of Nutrition, Indian Council of Medical Research, Hydrabad, India and approved by the Animal Ethical Committee, Annamalai University, Tamilnadu, India. Dexamethasone sodium phosphate and insulin were purchased from Sigma-Aldrich. Coconut oil and Indian vanaspati [brand name DALDA] were purchased from the local market, Chidambaram, India. All the other chemical and biochemical used for the experiments were of analytical grade.

Preparation of fat emulsion: Fat emulsion was prepared according to the method described [45] with some modifications. A constant volume of 100 ml fat emulsion containing 40 ml vanaspati, 30 ml coconut oil, 10 ml Tween 80, 5 ml propylene glycol and 15 ml distilled water, this is stored at 4 °C. This was considered as high-fat emulsion in this study and it was shaken before every use to ensure homogeneity.

### Experimental design

The animals were divided into 6 groups of 12 rats each and housed 6 rats per cage.

Group I [Control]: Rats given laboratory diet.

Group II [HF]: Rats were given 5 ml of high-fat emulsion.

Group III [HF-LD]: Rats were given 5 ml of high fat emulsion and Dex at a dose of 50 μg/kg/day.

Group IV [HF-HD]: Rats were given 5 ml of high-fat emulsion and Dex at a dose of 250 μg/kg/day.

Group V [LD]: Rats were given Dex, at a dose of 50 μg/kg/day.

Group VI [HD]: Rats were given Dex, at a dose of 250 μg/kg/day.

Dex dissolved in saline was administered subcutaneously [41]. The oral feeding of high-fat or water was started gradually from 1 ml to 5 ml in the first five days, to allow the rats to adapt to the high-fat feeding, and fixed at 5 ml on the fifth day of the experiment and the same is continued daily throughout the experiment, but the Dex administration was started from the first day of fat feeding onwards. Rats not receiving high-fat were given equal volume of drinking water using intra-gastric tube and rats not receiving Dex were administered equal volume of normal saline. All the rats were observed daily for the presence any clinical symptoms. Food intake, water intake and body weight changes were observed during the course of the experiment. Urine output was measured by collecting the urine in metabolic cages; the urine was collected under a layer of toluene. On the fourteenth day of the experiment the rats in the HF-HD groups were presented with severe glycosuria and polyuria. The glycosuria was confirmed by Benedict's qualitative test. Following the observation of glycosuria on the 14^th ^day of Dex treatment in the HF-HD rats, OGTT and euglycemic clamp were conducted on the subsequent days.

#### Oral Glucose Tolerance Test

After 14 days of Dex administration [i.e, on 15^th ^day], the rats were fasted overnight and OGTT was conducted in six rats from each group, by administering glucose [3 g/kg] with intra-gastric tube. Blood samples were obtained from tail vein at 0, 30, 60, 90, and at 120 min after glucose administration for the estimation of insulin and glucose. Insulin was determined by rat insulin enzyme linked immonosorbant assay [ELISA, kits, LINCO laboratories] and glucose was determined by assay kit [Erba diagnostics, Mannheim, Germany]. An "insulinogenic index", defined as the ratio of the change in circulating insulin to the change in the corresponding glycemic stimulus [46] was calculated using the equation, [30-min plasma insulin – Fasting Plasma Insulin]/[30-min plasma glucose – Fasting Plasma Glucose], the final unit is expressed as pmol/mmol [47].

#### Euglycemic clamp study

The remaining six rats from each group were used for euglycemic clamp study. The rats were anaesthetized by giving intra peritoneal injection of amobarbital sodium [25 mg/kg]. Under anesthesia, euglycemic clamp was conducted by cannulating in the jugular vein for the infusion of glucose and insulin. 10% glucose and insulin 1 IU/ml was administered [48] to keep the blood glucose in a steady state [140 mg/dl], the rate of glucose infusion was continuously adjusted while the insulin infusion was kept constant. Glucose infusion rate [GIR] [mg/kg/min] was measured under homeostasis five times during the experiment at 15 minutes intervals. Glucose levels were determined by glucometer [One touch-horizon-Lifescan. Inc.] [49]. Finally the rats were sacrificed by cervical decapitation and blood drawn for the separation of plasma. Plasma was stored at -80°C till the analysis. Total cholesterol [T.Cho] and triglycerides [Tgl] were estimated by assay kits [Erba diagnostics, Mannheim, Germany]. And high density lipoprotein cholesterol [HDL] was analyzed in the supernatant obtained after precipitation of the plasma with phosphotungstic acid/Mg2^+^. Plasma low-density lipoprotein cholesterol [LDL] was calculated from total cholesterol, HDL and Tgl values using the Friedwald equation [50] Plasma free fatty acid concentration was estimated by the method of Falholt *et al. *[51]. Statistical analysis was done by analysis of variance [ANOVA] followed by Duncan's multiple range test by means of the SPSS version 9.0 for Windows. P" value of less than 0.05 was considered to be statistically significant.

## Results

**Figure 1:**
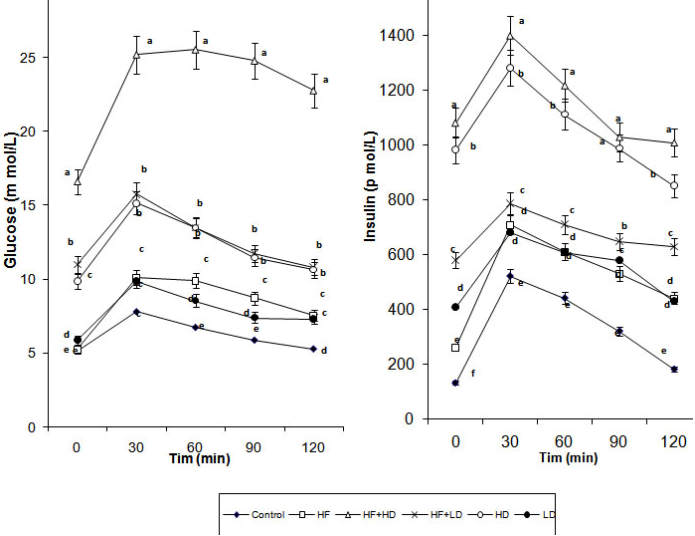
Changes in glucose and insulin values during OGTT of control and experimental groups. Values are mean ± of 6 experiments in each group. Values not sharing common superscript differ significantly at P ≤ 0.05.

Administration of Dex [both low and high dose] to high-fat fed rats [HF-HD and HF-LD] have caused severe hyperglycemia, hyperinsulinemia, altered lipid profile, reduced food intake, decreased insulinogenic index and GIR compared to the corresponding Dex [HD and LD] or high-fat alone treated rats [Fig. 1 and Table 1, 2 &3]. The effects were more pronounced and frank hyperglycemia with glycosuria [urine sugar 2+ and above] was noted in all the HF-HD rats on the 14^th ^day of the Dex treatment.

**Table 1:** Changes in the food intake and water intake of control and experimental animals.

	Control	HF	HF-HD	HF-LD	HD	LD
Food intake (g/Week)	112.5 ± 8.5^a^	89.7 ± 6.5^c^	20.5 ± 1.5^e^	70.0 ± 5.6^d^	99.5 ± 7.5^b^	113.5 ± 8.6^a^
Water intake (ml/24 h)	31.5 ± 2.34^a,b^	31.01 ± 2.1^a,b^	114.56 ± 8.39^d^	36.29 ± 3.40^c^	34.60 ± 4.28^b,c^	30.50 ± 5.10^a^

Values are mean ± SD of 6 experiments in each group. Values not sharing common superscripts differ significantly at P ≤ 0.05.

**Table 2:** Changes in the Insulinogenic index and GIR or control and experimental rats

	Control	HF	HF-HD	HF-LD	HD	LD
GIR (mg/kg/min)	24.50 ± 1.85^a^	17.85 ± 1.29^b^	2.85 ± 0.02^f^	8.85 ± 0.64^e^	10.98 ± 0.79^d^	14.87 ± 1.08^c^
Insulinogenic index (pmol/mmol)	139.30 ± 10.13^a^	94.34 ± 6.87^b^	30.52 ± 2.21^f^	44.52 ± 3.24^e^	56.81 ± 4.12^d^	68.54 ± 4.9^c^

Values are mean ± SD or 6 experiments in each group. Values not sharing common superscripts differ significantly at P ≤ 0.05.

**Table 3:** Changes in the lipid profile of control and experimental animals

	Control	HF	HF-HD	HF-LD	HD	LD
FFA (mmol/l)	0.29 ± 0.02^f^	0.81 ± 0.03^d^	3.91 ± 0.29^a^	1.61 ± 0.11^b^	1.41 ± 0.99^c^	0.62 ± 0.04^e^
Tgl (mmol/l)	1.48 ± 0.08^e^	3.26 ± 0.13^c^	7.69 ± 0.80^a^	4.41 ± 0.9^b^	3.29 ± 0.28^c^	2.94 ± 0.18^d^
T.Cho (mmol/l)	2.12 ± 0.13^f^	4.42 ± 0.25^c^	5.68 ± 0.21^a^	4.96 ± 0.21^b^	3.85 ± 0.19^d^	3.08 ± 0.29^e^
LDL (mmol/l)	0.71 ± 0.02^e^	2.98 ± 0.07^b^	3.63 ± 0.09^a^	3.51 ± 0.08^a^	2.51 ± 0.88^c^	1.75 ± 0.06^d^
HDL (mmol/l)	1.11 ± 0.04^a^	0.78 ± 0.38^b^	0.51 ± 0.38^e^	0.63 ± 0.41^d^	0.68 ± 0.31^c^	0.75 ± 0.28^b^

Values are mean ± SD or 6 experiments in each group. Values not sharing common superscripts differ significantly at P ≤ 0.05.

Administration of high-fat [HF] or higher dose of Dex [HD] decreased the food intake significantly when compared to control rats, but it was unaltered in the low dose Dex treated [LD] rats. When compared to HF and HD rats, the reduction in food intake was more pronounced in the HF rats. Administration of Dex together with the high-fat [HF-HD & HF-LD] reduced the food intake further when compared to the corresponding Dex alone treated [HD & LD] or HF alone treated rats. The reduction in food intake was more significant in the HF-HD rats compared to HF-LD rats [Table 1]. Body weight was reduced significantly after 14 days of Dex treatment in the HF-HD rats [data not shown] while no significant differences were observed in the other groups compared to control. The urine output was increased three fold in the HF-HD rats compared to control. The water intake remained unaltered in the HF and LD rats when compared to control, but it was increased significantly in the HD, HF-LD and HF-HD rats. The increase in water intake was more pronounced in the HF-HD rats compared to other groups [Table 1].

Figure 1 shows the glucose and insulin values during OGTT. Fasting glucose was not altered between control and HF rats, but it was increased significantly in the HD and LD rats and the increase was directly proportional the dose of Dex used. The dose dependent effect of Dex on fasting glucose was further increased when the same dose was given to high-fat fed [HF-HD and HF-LD] rats compared to corresponding Dex [HD & LD] or HF alone treated rats [Fig. 1]. Polyuria with urine sugar more than 2+ was noticed in all the HF-HD rats while no such observations were made on any other groups. The criteria for diabetic type are a peak plasma glucose concentration > 16.8 mmol or 120-min post load plasma glucose concentration > 11.2 mmol. According to this criterion, HF-HD rats become severe diabetic on the 14th day of treatment [52].

Although the fasting glucose was not increased in the HF rats the fasting insulin was increased significantly in the HF rats compared to control. Dex treatment increased significantly the fasting insulin values in the HD and LD rats compared to control, the increase was directly proportional to the Dex dose [Fig. 1]. When compared to HF rats the fasting insulin was increased more significantly in the Dex treated [HD & LD] rats. The dose dependent increase in fasting insulin was further increased when the same dose of Dex was administered to high-fat fed [HF-HD and HF-LD] rats [Fig. 1].

Administration of high fat [HF] or Dex [HD & LD] to Wistar rats decreased the insulin sensitivity as shown by reduced GIR, and reduced the insulin response, as shown by reduced insulinogenic index, significantly when compared to control rats. The decrease in insulinogenic index and GIR were more significant in the Dex [HD & LD] treated rats compared to HF rats. Administration of Dex dose dependently decreased insulinogenic index and GIR in the [HD & LD] rats, but the same dose further decreased the GIR and insulinogenic index when administered to high-fat fed rats [HF-HD & HF-LD] [Table 2].

High-fat [HF] feeding induced severe hyperlipidemia as shown by increased FFA, Tgl, T.Cho, LDL and decreased HDL when compared to control rats. Dex treatment also increased the hyperlipidemia as shown by increased FFA, Tgl, T.Cho, and LDL and reduced HDL in the HD and LD rats compared to control. When compared between HF and Dex treated [HD & LD] rats, FFA was increased, Tgl was unaltered, HDL decreased in the HD rats, and all other values were increased in HF rats [Table 3]. Dex dose dependently alerted the lipid profile values [HD and LD], but the same dose altered the lipid values further when it was administered to high-fat fed [HF-HD and HF-LD] rats [Table 3].

## Discussion

Administration of Dex has been reported to induce insulin resistance and hyperglycemia in animal studies [41,42]. Our results shows that the hyperglycemic effect of Dex have been potentiated/aggravated when it was administered to high-fat fed [HF-HD and HF-LD] rats compared to Dex alone [HD or LD] treated rats. The individual effects of Dex [HD or LD] or high-fat [HF] is very minimal with respect to hyperglycemia, hyperinsulinemia, hyperlipidemia, GIR and insulinogenic index when compared to rats co-administered with Dex and high-fat [HF-HD or HF-LD] [Table 1, 2,3 and Figure. 1].

Reports have shown that high-fat fed animal tend to eat less compared to rats fed normal diet [53,54]. We have also found a reduction in food intake in the HF rats compared to control [Table 1]. Glucocorticoids are reported to have dual metabolic action on food intake and body weight gain which depends on the dose used [55]. At high dose, it decrease the food intake and body weight, in contrast at lower dose it increase the appetite [42,55]. Similar findings were observed in the HD rats, while in the LD rats food intake was not increased but consumed the same amount as that of control, which might be due to the variations in the dose used.

Severe metabolic complications were observed in the HF-HD rats, which might be responsible for the loss of body weight and increased water intake observed in HF-HD rats compared to HD, HF-LD, LD, and HF rats [Table 2]. The food intake was reduced in the HD, HF-LD and HF rats, but body weight was not affected. High-fat feeding might have compensated the energy loss due to reduced food intake in the HF and HF-LD rats. In the HD rats, the metabolic complications are significantly less when compared to HF-HD rats, and the duration of the study is also very short to show any minimal changes in the body weight [Table 1]

Hyperlipidemic effect of high-fat feeding has been reported previously [45]. This is in accordance with our findings in the HF rats, where all the lipid profiles were altered significantly [Table 3]. Glucocorticoids have been reported to induce lipolysis [4,42] causing hyperlipidemia. We have observed a similar finding in our study in the Dex treated [HD or LD] rats [Table 3]. Glucocorticoids have been reported to induce hyperlipidemia by inhibiting lipoproteins lipase and altering the level/action of hormone sensitive lipase [56,57] thus it disable the uptake of FFA by adipose tissue [58]. It have been reported previously that in the presence of high-caloric diet, cortisol affect lipid metabolism adversely [59]. In the HF-HD and HF-LD rats, due the hyperlipidemic effect of Dex, the absorbed lipid might not have been stored properly, which might have leads to increased lipids levels in the circulation [Table 3].

Glucocorticoids are reported to mediate their effects differentially on peripheral vs. central organs, depending on their concentrations [60]. The effect of Dex have been reported to be increased when it is administered intracerebroventricularly compared to the same administered intraperitoneally and the availability of Dex in the central nervous system is crucial for its effect than its presence in the peripheral circulation [61]. At the central level, glucocorticoids are reported to affect neuronal pathways involved in the regulation of food intake and energy expenditure by modulating the hypothalamic neuropeptides [59,61] at the periphery, they modulate metabolic pathways. It was also reported to influence the leptin-insulin axis [42,62]. The exact mechanism of reduction in food intake in the HF-HD and HF-LD is not known, but the hyperlipidemia induced by high-fat feeding might have potentiated the action/availability of Dex in the central nervous system causing reduced food intake in the HF-HD and HF-LD rats.

Cortisol circulates in the blood in both free and bound forms; cortisol's biological half-life is around 80 min [40]. In plasma, cortisol is predominantly bound to corticosteroid-binding globulin, with a small amount bound loosely to albumin, and the remainders free [40]. The remaining free cortisol molecule is lipophilic and has a low molecular weight [MW ~362 Da], passing from capillaries into tissues mainly by passive diffusion [40]. The intracellular and central nervous system availability of glucocorticoids are controlled by P-glycoprotein [Pgb] and cortisol binding globulin [CBG] [63,64]. Dietary free fatty acids have been reported to alter the availability of glucocorticoids by modulating CBG [64]. CBG is usually saturated in the upper physiological range of cortisol concentrations, so that the response of CBG to increasing cortisol level [as observed in Cushing's syndrome and iatrogenic anti-inflammatory treatment] is non-linear and have no role in these situations [24]. The effect of increased level of free fatty acid on Pgp and its regulation of stereoid transport to central nervous system are not known. It is possible that the hyperlipidemia observed in the HF-HD and HF-LD rats might have altered the steroid-proteins [CBG & Pgp] interactions which in turn might have altered the availability of Dex.

Physical state of the membrane is proposed to play major role in the expression of several genes involved in ageing & diseases [65]. Fatty acid composition in the diet is one of the determining factors of membranes' physical state. Higher amount of saturated fatty acid is reported to alter the fatty acid composition of membrane, which in turn affect its fluidity and permeability [18,66]. Hyperlipidemia is also proposed to alter the membrane lipid composition and its physical state [fluidity], which are decisive factors in the process of perception and signal transduction which trigger the expression of several genes [67]. Moreover, in a normolipidaemic plasma profile, the distribution of triglycerides, cholesterol and proteins is optimal for the dissemination of hydrophobic lipids through the aqueous milieu [36]. When this profile is perturbed, the resulting changes are not only detrimental to the health of an individual, but also provide an altered environment for drug transport and delivery. Any alteration in drug-protein interactions might shift the proportion of the drug that is available to the tissues, and potentially affect drug efficacy [36]. And for transport processes to function efficiently either proper fluidity of the membrane or correct lipid environment or both are necessary [67-69].

Lipophilic drugs have a greater tendency to dissolve in the lipid of the membrane than in the aqueous media bathing it [37]. There is a linear relationship between the lipophilicity of drugs and their brain penetration [70,71]. It have been stressed for lipophilic drugs that the membrane drug volume is to be considered instead of "free" and "bound" concentrations of the drug in the circulation [72]. Apart from these, steroid hormones are also reported to alter the structured lipid systems even at physiological concentrations [73,74] and unbound steroids are transported across the blood-brain-barrier to enter into the brain [70,71]. Several drugs such as chlorpromazine, dibucaine, lignocaine, imipramine, tetracaine and procaine, which are used as psychotropic drugs or local anaesthetics, are reported to cause membrane deformation or fluidization [65].

Further, any changes in lipid profile [caused by obesity, diet, weight gain, medication or illness] [35-37] values have been reported to alter the pharmacological of activity of lipophilic drugs. Pharmacological action of the lipophilic drug clozapine was reported to be potentiated by hyperlipidemia. The central nervous system availability of this drug was reported to be increased by hyperlipdiemia, facilitating its penetration across the blood brain barrier [35]. We have used coconut oil and vanaspati to induce hyperlipidemia and they are a good source of saturated and trans fatty acids. Severe hyperlipidemia was also observed in the HF-HD and HF-LD rats compared to Dex alone treated rats [Table 3]. Dex is also a lipophilic hormone. All these factors might have facilitated the increased cellular and central nervous system availability/action of Dex which in turn might have caused reduced food intake and metabolic complications in the HF-HD and HF-LD rats.

Although no direct evidence for the potentiating effect of hyperlipidemia on Dex in the HF-HD and HF-LD rats is available, Dex clearance was reported to be increased in patients taking carbamazapine [75]. Carbazapine treatments reported to alter the lipid metabolism and cause hyperlipidemia in patients [75,76]. No data was available correlating the Dex clearance and increased hyperlipidemia in these patients.

Reports have shown that the administration of high-fat induce insulin resistance, which is reported to be mediated by the high level of circulating FFA [77,78]. Similar findings were observed in the HF rats where the GIR and insulinogenic index were reduced while lipid values were increased significantly compared to control rats [Table 2 &3]. It have been reported that the high-fat alone, although induce insulin resistance, could not produce diabetes [77,20]. It have also been reported that the role of free fatty acid in the development of diabetes is a secondary phenomenon [20]. Our study also shows that fourteen days of high-fat feeding decreased the insulin sensitivity [reduced GIR] and insulinogenic index minimally when compared to Dex treated [HD and LD] rats.

Administration of Dex has been reported to induce insulin resistance and hyperglycemia in several animal studies [41,42]. Similar effects were observed in the HD and LD rats where the GIR and insulinogenic index were reduced significantly [Table 2] compared to HF and control rats. Although the hyperlipidemia was increased significantly in the HF rats when compared LD rats, the diabetogenic effect were more pronounced in the LD rats. It have been reported that lipophilic molecules can passively diffuse across cell membranes a process that is driven by the concentration gradient [40]. The increased level of Dex in the HD and LD rats might have altered the membrane structure and enter the cell by concentration gradient which in turn have caused insulin resistance and impaired glucose tolerance in the HD and LD rats [Fig. 1 & Table 2] [70,71,73,74]. The results in the HD and LD rats can be correlated to exogenous or endogenous elevation of cortisol, such as observed in Cushing syndrome.

Increased clearance and intracellular action/availability of cortisol have been reported in obesity [24,26,27]. The increased action/availability of cortisol was proposed in the pathogenesis of insulin resistance, metabolic syndrome and type 2 diabetes [23,26-28]. Administration of hydrocortisone to centrally obese patients reported to induce severe insulin resistance compared to normal counterparts [79]. In an animal study administration of a massive doses of Dex [5 mg/kg for 24 days] to Wistar rats induced diabetes only in < 20% rats, but in obese Zucker [fa/fa] rats diabetes was induced in 100% of animals with a dose of Dex only 4–8% of the used in Wistar rats [80]. In another study, reduction of fat/calorie have been reported to reduce the insulin resistance caused by dexamethasone in rats [39]. Hyperlipidemia has been reported to be associated with obesity, metabolic syndrome and its related disorders. From the results obtained in the HF-HD and HF-LD rats, it can be speculated that the hyperlipidemia might have potentiated the action/availability of normal or mildly elevated cortisol causing metabolic disorders.

It is further evidenced by the following studies that Knock out [81] or pharmacological inhibition of the enzyme 11β-HSD1 [82] or rats treated with antiglucocorticoids are protected from the high-fat diet induced insulin resistance [83]. These reports indicate that cortisol action is very essential for high-fat feeding to mediate its metabolic complications. On the other hand, lowering of free fatty acid in the circulation reported to prevent the diabetogenic effect of cortisol [84,85]. Although both cortisol and dyslipidemia might be involved in the diabetogenic process, cortisol might be the primary diabetogen, the associated/induced hyperlipidemia might involve in the diabetogenic process by facilitating the intracellular availability/action of Dex. Elevated cortisol also reported to increase the hyperlipidemia [56-58], once elevated it may interact with cortisol forming a vicious cycle.

Asians are quite prone to visceral fat accumulation and have greater waist circumferences than Europeans [86] and are prone to develop type 2 diabetes [87]. Altered cortisol metabolism and its aggravation by obesity have been implicated in the development of metabolic complications in these populations [88]. Poly cystic ovary syndrome is a most common endocrinological disorder of reproductive age women, 50% of the afflicted women are obese [89]. Its etiology remains unknown, a variety of theories such as defect at the level of the hypothalamus/pituitary and alteration in the lipid metabolism have been proposed [89,90]. From our results we can speculate that either increased cortisol level or altered lipid metabolism might be the initiating factor of the disease, but it might be further aggravated by the interaction between the two, forming a vicious cycle.

Though PCOS affect both obese and lean women, the metabolic complications are reported to be comparatively less in the lean women compared to obese [10]. Although altered function 11 β-HSD1 have been implicated to explain the difference [10], the role of altered lipid profile in the obese women compared to lean might have interacted with available cortisol when compared to lean PCOS patients, in whom the alterations of lipids reported to be less comparatively[89].

Gestational diabetes is also associated with wide variations in lipid and cortisol metabolism [9] the interaction between altered lipid and cortisol might play a role in the pregnancy induced hyper glycemia. Altered lipid and cortisol metabolism is also associated in several animal models of metabolic disorders such as db/db, ob/ob mice and obese Zucker rats [91-93]. *Psammomys obesus*, the desert rat is a well-defined animal model for dietary induced insulin resistance and type 2 diabetes [94]. Seasonal variation cortisol has been reported in these rats [95] but no such seasonal variations were reported in the non-seasonal Wistar rat, in which the ordinary diet will not induce diabetes [77]. The interaction between cortisol and lipid metabolism might play a role in the metabolic complications observed in these rats when fed laboratary diet.

Chronic stress has been reported to promote the consumption of high-caloric food and obesity [96]. Variations in the macronutrient composition of the diet, such as high-fat, is reported to affect mood and neuroendocrine response to stress [30,97]. Recent report have shown that macronutrient content of the diet alter the extraadrenal regeneration of cortisol [30]. High-energy, particularly high-fat is reported as a backround form of chronic stress that elevates cortisol levels. And the high level of cortisol reported to favor the increased food intake, and at their periphery alter lipid metabolism forming a vicious cycle [59,98]. From our results and also from the available information, we can speculate that the increased availability of lipid in the circulation potentiate the action/availability of cortisol which in turn causes its adverse effects. Similarly the elevated level of cortisol also might have altered the lipid metabolism, especially during positive caloric balance forming a vicious cycle.

Administration Dex have been reported induce several features characteristics of the neurohormonal response to stress [39] and it have been correlated to the stress of critically ill patients [39]. It have been reported that "it is better to err on the side of giving too few than too many calories to patients because, it is likely that infectious and metabolic complications are increased by overfeeding [99]. When the administration of Dex have been correlated to the stressful situations of critically ill patients [39] the same can be extended to other chronic stressful situation which alter the cortisol secretion. And, though the amount of fat administered is very high in this study compared to the human consumption it can be considered as a model for positive caloric balance. Then the outcome of the results observed in the HF-HD and HF-LD rats indicates that the effect of persistent stress [8] during positive energy balance [3] might have a pathological role in the development insulin resistance and its related complications.

## Conclusion

The administration of dexamethasone to high-fat fed rats have caused severe insulin resistance, hyperglycemia and hyperlipidemia dose dependently compared to rats treated dexamethasone of high-fat alone. Hyperlipidemia induced by high fat feeding might have facilitated the intracellular availability/action of dexamethasone probably by altering the membrane fluidity and permeability and also transport proteins, which in turn might have caused the insulin resistance and hyperglycemia. High-fat and dexamethasone induced insulin resistance can be used as a new model to study the pathogenesis of type 2 diabetes. As such this study is first and preliminary of its kind and further studies are warranted.

## Abbreviations

FFA: Free fatty acids; HPA-axis: Hypothalamo-Pituitary axis; 11 βHSD1: 11β-hydroxysteroid dehydrogenase 1; Dex: Dexamethasone; OGTT: Oral glucose tolerance test; HF: High-fat treated; HF-LD: High-fat + Low-dose of Dex; HF-HD: High-fat + High-dose of Dex; LD: Low-dose of Dex; HD: High-dose Dex; GIR: Glucose infusion rate; T.Cho: Total Cholesterol; Tgl: Triglycerides; HDL: High-density lipoprotein cholesterol; LDL: Low-density lipoprotein cholesterol; ANOVA: Analysis of variance; Fig: Figure; CBG: cortisol binding globulin; PCOS: Polycystic ovarian syndrome; Pgb: P-glycoprotein.

## Competing interests

The authors declare that they have no competing interests.

## Authors' contributions

SS contributed to conception and design, analyzed the data and drafted the manuscript, SR contributed to data analysis and critically reviewed the manuscript for intellectual content, VPM designed and managed the overall study, interpretation of the results and critically reviewed the manuscript for intellectual content. All authors read and approved the final manuscript.

This study was not aided by any grants or funding agencies.
